# Distinct characteristics of distant metastasis in early-onset gastric cancer patients compared to late-onset patients: An observational study

**DOI:** 10.1097/MD.0000000000038098

**Published:** 2024-05-17

**Authors:** Feng Wu, Shuang Wu, Shujie Shuai

**Affiliations:** a Department of Gastroenterology, Jingdezhen Second People’s Hospital, Jingdezhen City, Jiangxi Province, China; b Department of General Surgery, The Fifth People’s Hospital of Jingdezhen City, Jingdezhen City, Jiangxi Province, China.

**Keywords:** early-onset gastric cancer, Lauren classification, liver metastasis, SEER

## Abstract

Presently, there is limited understanding of the features of distant metastasis in early-onset gastric cancer (GC). To explore these disparities, a retrospective study utilizing the Surveillance, Epidemiology, and End Results (SEER) database was undertaken. The SEER database was utilized to extract patient data, and multivariate logistic regression analysis was employed to identify the risk factors associated with distant metastasis and liver metastasis. Propensity score matching (PSM) was used to compare the occurrence of liver metastasis among patients based on their age at diagnosis. The study included 2684 early-onset GC patients and 33,289 late-onset GC patients. Preliminary data analysis indicated that early-onset GC patients exhibited more aggressive characteristics such as poor cell differentiation, advanced T stage, and a higher incidence of distant metastasis, excluding liver metastasis. Multivariate logistic regression analysis identified younger age as an independent risk factor for distant metastasis, along with T stage, lymph node metastasis (LNM), and tumor size (>3 cm). Another regression analysis revealed that younger age, diffuse type, and female gender were protective factors against liver metastasis. Through PSM, 3276 early-onset GC patients were matched with an equal number of late-onset GC patients, revealing that patients with early-onset GC had fewer instances of liver metastasis but a higher prevalence of distant metastasis. Our findings suggest that early-onset serves as a protective factor against liver metastasis in GC, while it poses a risk for distant metastasis, likely influenced by the increased prevalence of diffuse-type GC in early-onset patients.

## 1. Introduction

Gastric cancer (GC) is 1 of the most common cancers worldwide, characterized by a high rate of cancer-related mortality.^[1]^ The classification of GC primarily encompasses 2 types: histological classification and gross classification. Commonly utilized classifications include Borrmann typing, Lauren typing, and the WHO classification.^[2]^ The Lauren typing system is predominantly favored in clinical practice due to its association with prognosis and treatment strategies. This system categorizes GC into diffuse type, intestinal type, and mixed type, thus guiding clinicians in determining appropriate management approaches.^[2]^ Numerous studies have highlighted the contrast in clinical characteristics between diffuse-type and intestinal-type GC.^[3]^ For example, patients with diffuse-type GC frequently exhibit a higher propensity for lymph node metastasis (LNM) and experience a less favorable prognosis compared to those with the intestinal type.^[3]^ Early diagnosis plays a crucial role in improving outcomes for patients with GC. Additionally, advancements in medical imaging techniques and biomarker research may aid in early detection, thus enhancing prognosis and quality of life for patients undergoing surgery.^[2]^ The statistic highlighting that over 40% of GC patients are diagnosed at an advanced stage underscores the need for increased efforts in early detection and screening programs.^[4]^ The 5-year survival rate for GC remains dishearteningly low, with a value of <25%.^[4]^ Greater awareness among healthcare providers and the general public about the risk factors and symptoms of GC could lead to earlier diagnosis, potentially improving treatment outcomes and overall prognosis for patients.

Actually, the incidence of early-onset GC which was defined according to diagnosed age (<50 years older, younger GC) is stably rising.^[5]^ Furthermore, owing to their younger age, many patients are prone to overlooking the symptoms of GC. Recent research has shed light on this concerning trend among younger individuals with GC, revealing that they may present with more advanced stages, including distant metastasis, leading to a poorer prognosis. Understanding these nuances and conducting targeted studies can help improve early detection, treatment strategies, and outcomes for this demographic group.^[6]^ Research indicates a worrying trend in GC cases, with the proportion of cases presenting with distant metastasis rising from 25% in 2005 to 44% in 2019. The most common sites of metastasis include the liver, distant lymph nodes, and peritoneum.^[7]^ The liver stands out as the primary site of distant metastases, affecting approximately 50% of GC patients. Despite this, the relationship between age and distant metastasis has yet to be fully elucidated. The specific characteristics of distant metastasis in early-onset versus late-onset patients remain unexplored, highlighting the need for further research to better understand these distinctions and tailor treatment approaches accordingly.

In this study, we aimed to examine the characteristics of distant metastasis and risk factors in GC patients across different age groups at diagnosis. We analyzed data from 36,973 patients in the SEER database, categorizing them into early-onset GC (<50 years old) and late-onset GC (≥50 years old) groups. Through logistic regression analysis, we identified key clinical factors influencing distant metastasis. By employing propensity score matching (PSM) methods, we balanced baseline information and compared the differences in distant metastasis between the 2 age cohorts. Our results revealed that early-onset patients with GC exhibited a higher likelihood of distant metastasis, although they had fewer cases of liver metastasis compared to late-onset patients.

## 2. Methods

### 2.1. Patients

Our study cohort comprised GC patients extracted from the SEER database using the National Cancer Institute’s SEER*Stat software (version 8.4.2). Ethical approval for our study was obtained in compliance with regulations set forth by the SEER ethics committee. Tumors were categorized into diffused type and intestinal-type based on the International Classification of Diseases in Oncology (ICD-O-3) codes, including codes 8145, 8142, 8490, and 8010 for diffuse type, and codes 8211, 8140, and 8144 for intestinal-type.^[8]^ In our study, patients were selected based on the following criteria: individuals above 20 years old diagnosed with GC confirmed by positive histology between 2010 and 2020; and the patients with complete and detailed diagnostic codes. All eligible patients meeting these criteria were included in the study without any exclusions.

### 2.2. Clinicopathological factors

In our study, clinicopathological variables extracted from the SEER database comprised age, race, sex, T stage (8th edition TNM staging system), M stage, tumor size, N stage (8th edition TNM staging system), cell differentiation, distant metastasis, survival time, and status. Patients were divided into 2 groups based on age: early-onset and late-onset, with a cutoff value of 50 years old. We further investigated the association between age and liver metastasis by subdividing age groups into 20 to 29 years, 30 to 39 years, 40 to 50 years, 50 to 59 years, 60 to 69 years, and over 70 years. Race was categorized into White, Black, other, and unknown. Sex was classified as male and female. T stage included Tis, T1, T2, T3, T4, and unknown. N stage was recorded as N1 (Yes), N2 (Yes), N3 (Yes), N0 (No), and unknown. M stage was detailed as negative (No), positive (Yes), and unknown. Tumor size was grouped into <3 cm, ≥3 cm, and unknown categories. GC type was characterized by Lauren classification into diffused type and intestinal type. Cell differentiation encompassed well, moderate, poorly, undifferentiated, and unknown subgroups. The site of distant metastasis served as the primary observation indicator, inclusive of metastases to the bone, brain, liver, lung, and other sites. Due to the fact that liver metastasis occupies the majority of the data, and analysis indicates that it has a different relationship with age, it was singled out for separate analysis.

### 2.3. Statistical analysis

In our study, patients were segregated into 2 groups: early-onset and late-onset GC, with Pearson Chi-squared test employed to explore associations among categorical variables. Missing or unknown values were retained in the dataset as deleting or interpolating them could potentially distort real-world data. To identify potential risk factors for metastasis, we conducted multivariate logistic regression analysis and presented the results as odds ratios (OR) with corresponding 95% confidence intervals (CI). In our manuscript, we found the basic information such as sex, race, tumor site, grade, T stage, and N stage were not balanced. Addressing the imbalance between the early-onset and late-onset GC groups, we utilized PSM via the MatchIt package in R software to obtain matched data. We set the caliper value at 0.01 and assessed balance through *P* values and standardized mean differences (SMD). Balance was deemed achieved when SMD was below 0.1 and *P* values exceeded .05.^[9]^ The comprehensive process involved several steps. Initially, we computed propensity scores for each patient using a multivariate logistic regression model. Subsequently, patients were matched in a 1:1 ratio between the early-onset and late-onset groups. We then assessed and illustrated differences in all variables based on *P* values and standardized mean differences (SMD). Lastly, we examined correlations using the Chi-squared test. All statistical analyses were carried out using R software (version 4.3.2, StataCorp LLC, College Station, Texas). Results were deemed statistically significant when *P* values were below .05.

## 3. Results

### 3.1. Basic information about included patients

In this study, we utilized the SEER database – a publicly available project initiated by the National Cancer Institute and the Department of Population Science in the United States to monitor epidemiology and survival outcomes and enroll patients according to our objectives. As shown in Table 1, we included a total of 36,973 patients based on pathological diagnoses from 2010 to 2020. Of these, 3684 patients were in the late-onset group and 33,289 patients were in the late-onset group. It was observed that the late-onset group had a slightly higher percentage of White patients compared to the early-onset group (70.6% vs 68.6%), although the difference was not statistically significant (*P* < .05).

**Table 1:** Basic information about included patients diagnosed from 2010 to 2020.

	Overall	Early-onset GC	Late-onset GC	*P* value
n	36,973	3684	33,289	
Race (%)				.001
White	26,044 (70.4)	2526 (68.6)	23,518 (70.6)	
Black	4442 (12.0)	450 (12.2)	3992 (12.0)	
Other	6243 (16.9)	668 (18.1)	5575 (16.7)	
Unknown	244 (0.7)	40 (1.1)	204 (0.6)	
Sex (%)				<.001
Male	23,645 (64.0)	2128 (57.8)	21,517 (64.6)	
Female	13,328 (36.0)	1556 (42.2)	11,772 (35.4)	
Site (%)				<.001
Cardia	12,463 (33.7)	959 (26.0)	11,504 (34.6)	
Fundus	1321 (3.6)	125 (3.4)	1196 (3.6)	
Body	3371 (9.1)	420 (11.4)	2951 (8.9)	
Antrum	6655 (18.0)	633 (17.2)	6022 (18.1)	
Pylorus	955 (2.6)	79 (2.1)	876 (2.6)	
Lesser curvature	2662 (7.2)	265 (7.2)	2397 (7.2)	
Greater curvature	1230 (3.3)	158 (4.3)	1072 (3.2)	
Overlapping	2712 (7.3)	342 (9.3)	2370 (7.1)	
Unknown	5604 (15.2)	703 (19.1)	4901 (14.7)	
Grade (%)				<.001
Well-differentiated	1288 (3.5)	65 (1.8)	1223 (3.7)	
Moderately differentiated	7672 (20.8)	407 (11.0)	7265 (21.8)	
Poorly differentiated	18,371 (49.7)	2299 (62.4)	16,072 (48.3)	
Undifferentiated	466 (1.3)	52 (1.4)	414 (1.2)	
Unknown	9176 (24.8)	861 (23.4)	8315 (25.0)	
T_stage (%)				<.001
Tis	77 (0.2)	10 (0.3)	67 (0.2)	
T1	8383 (22.7)	595 (16.2)	7788 (23.4)	
T2	2962 (8.0)	249 (6.8)	2713 (8.1)	
T3	8103 (21.9)	820 (22.3)	7283 (21.9)	
T4	5896 (15.9)	839 (22.8)	5057 (15.2)	
Unknown	11,552 (31.2)	1171 (31.8)	10,381 (31.2)	
N_stage (%)				<.001
No	15,149 (41.0)	1269 (34.4)	13,880 (41.7)	
N1	8482 (22.9)	962 (26.1)	7520 (22.6)	
N2	2809 (7.6)	340 (9.2)	2469 (7.4)	
N3	2755 (7.5)	381 (10.3)	2374 (7.1)	
Unknown	7778 (21.0)	732 (19.9)	7046 (21.2)	
M_stage (%)				<.001
No	22,083 (59.7)	1660 (45.1)	20,423 (61.4)	
Yes	13,207 (35.7)	1880 (51.0)	11,327 (34.0)	
Unknown	1683 (4.6)	144 (3.9)	1539 (4.6)	
GC type				<.001
Intestinal GC	27,608 (74.7)	1942 (52.7)	25,666 (77.1)	
Diffuse GC	9365 (25.3)	1742 (47.3)	7623 (22.9)	
Size				.011
<3 cm	6853 (18.5)	634 (17.2)	6219 (18.7)	
≥3 cm	13,967 (37.8)	1361 (36.9)	12,606 (37.9)	
Unknown	16,153 (43.7)	1689 (45.8)	14,464 (43.4)	
Bone_M (%)				<.001
No	32,681 (88.4)	3156 (85.7)	29,525 (88.7)	
Yes	1762 (4.8)	310 (8.4)	1452 (4.4)	
Unknown	2530 (6.8)	218 (5.9)	2312 (6.9)	
Brain_M (%)				<.001
No	34,141 (92.3)	3417 (92.8)	30,724 (92.3)	
Yes	261 (0.7)	43 (1.2)	218 (0.7)	
Unknown	2571 (7.0)	224 (6.1)	2347 (7.1)	
Liver_M (%)				<.001
No	28,835 (78.0)	2984 (81.0)	25,851 (77.7)	
Yes	5734 (15.5)	501 (13.6)	5233 (15.7)	
Unknown	2404 (6.5)	199 (5.4)	2205 (6.6)	
Lung_M (%)				<.001
No	32,381 (87.6)	3220 (87.4)	29,161 (87.6)	
Yes	1976 (5.3)	242 (6.6)	1734 (5.2)	
Unknown	2616 (7.1)	222 (6.0)	2394 (7.2)	
Other site_M (%)				<.001
No	29,853 (80.7)	2499 (67.8)	27,354 (82.2)	
Yes	5437 (14.7)	1041 (28.3)	4396 (13.2)	
Unknown	1683 (4.6)	144 (3.9)	1539 (4.6)	
Status (%)				<.001
Alive	13,083 (35.4)	1458 (39.6)	11,625 (34.9)	
Dead (GC)	17,260 (46.7)	1964 (53.3)	15,296 (45.9)	
Dead (other cause)	454 (1.2)	81 (2.2)	373 (1.1)	
Unknown	6176 (16.7)	181 (4.9)	5995 (18.0)	

In comparing the early-onset and late-onset groups, it was noted that the proportion of female patients increased while the proportion of male patients decreased significantly in the early-onset group (*P* < .001). Regarding tumor sites, there was a notable decrease in cardia-located tumors (26.0% vs 34.6%) and an increase in body-located tumors (11.4% vs 8.9%) in the early-onset group (*P* < .001). However, the change in the distribution of tumors in the antrum was not substantial.

Regarding cell differentiation, tumors in the early-onset group tended to be poorly differentiated (62.4% vs 48.3%), indicating more aggressive behavior in early-onset patients with GC. Additionally, the early-onset group had a higher percentage of tumors at T3/T4 stage (45.1% vs 37.1%) and with positive LNM (45.6% vs 37.1%) compared to the late-onset group. The proportion of diffuse GC in the early-onset group was more than double that of the late-onset group (47.3% vs 22.9%, *P* < .001).

In terms of tumor size distribution, due to a larger proportion of cases with unknown sizes, the differences in tumor size distribution were not significant. The rate of tumors with distant metastasis was notably higher in the early-onset group compared to the late-onset group (51.0% vs 34.0%). Further analysis revealed that rates of bone and brain metastasis were higher than in the early-onset group compared to the late-onset group (8.4% vs 4.4%, 1.2% vs 0.7%), with a higher rate of lung metastasis in the early-onset group (6.6% vs 5.2%), all showing statistical significance (*P* < .001). Conversely, the rate of liver metastasis was lower in the early-onset group compared to the late-onset group (13.6% vs 15.7%). The mortality rate due to GC in the early-onset group was higher than that in the late-onset group (53.3% vs 45.9%).

### 3.2. Identifying risk factors of distant metastasis

To examine the risk factors associated with distant metastasis, we excluded patients without information on distant metastasis and focused on 35,290 patients, including 13,207 patients with distant metastasis (as shown in Table 2). We conducted univariable and multivariable logistic regression analyses to pinpoint independent factors contributing to this outcome. Notably, late-onset GC patients exhibited a lower risk of distant metastasis compared to early-onset patients (OR = 0.52, *P* < .001). When considering race, it appeared that Black patients had a similar rate of distant metastasis compared to White patients, while individuals of other racial backgrounds showed a reduced risk of distant metastasis. Gender did not appear to have a significant association with the occurrence of distant metastasis. Analysis of tumor location revealed varying risks of distant metastasis. Tumors located in the fundus and body were more prone to distant metastasis compared to those in the cardia (OR > 1, *P* < .001), as were tumors in the greater curvature and overlapping areas. In contrast, tumors in the antrum, pylorus, and lesser curvature showed lower risks of distant metastasis (OR < 1, *P* < .001). Poorly differentiated tumors emerged as risk factors for distant metastasis compared to well-differentiated tumors (OR > 1, *P* < .001), while the rates of distant metastasis between diffuse and intestinal GCs appeared similar. Advanced tumor stage (T3/T4) and positive LNM were identified as risk factors for distant metastasis (OR > 1, *P* < .001). Additionally, larger tumor size was associated with an increased likelihood of distant metastasis (OR = 1.65, *P* < .001).

**Table 2:** Cross-sectional associations between other clinical factors and distant metastasis.

Dependent:		No (N = 22,083)	Yes (N = 13207)	OR (univariable)	OR (multivariable)
Age	Early-onset GC	1660 (7.5%)	1880 (14.2%)		
	Late-onset GC	20,423 (92.5%)	11,327 (85.8%)	0.49 (0.46–0.53, *P* < .001)	0.52 (0.48–0.56, *P* < .001)
Race	White	15,361 (69.6%)	9558 (72.4%)		
	Black	2723 (12.3%)	1610 (12.2%)	0.95 (0.89–1.02, *P* = .134)	0.97 (0.90–1.05, *P* = .500)
	Other	3843 (17.4%)	1971 (14.9%)	0.82 (0.78–0.88, *P* < .001)	0.84 (0.78–0.90, *P* < .001)
	Unknown	156 (0.7%)	68 (0.5%)		
Sex	Male	14,123 (64%)	8466 (64.1%)		
	Female	7960 (36%)	4741 (35.9%)	0.99 (0.95–1.04, *P* = .779)	
Site	Cardia	7669 (34.7%)	4211 (31.9%)		
	Fundus	728 (3.3%)	547 (4.1%)	1.37 (1.22–1.54, *P* < .001)	1.31 (1.15–1.50, *P* < .001)
	Body	1919 (8.7%)	1301 (9.9%)	1.23 (1.14–1.34, *P* < .001)	1.25 (1.13–1.37, *P* < .001)
	Antrum	4478 (20.3%)	1880 (14.2%)	0.76 (0.72–0.82, *P* < .001)	0.83 (0.77–0.90, *P* < .001)
	Pylorus	650 (2.9%)	248 (1.9%)	0.69 (0.60–0.81, *P* < .001)	0.77 (0.65–0.91, *P* = .003)
	Lesser curvature	1801 (8.2%)	738 (5.6%)	0.75 (0.68–0.82, *P* < .001)	0.80 (0.72–0.89, *P* < .001)
	Greater curvature	724 (3.3%)	452 (3.4%)	1.14 (1.01–1.29, *P* = .041)	1.27 (1.10–1.46, *P* = .001)
	Overlapping	1411 (6.4%)	1167 (8.8%)	1.51 (1.38–1.64, *P* < .001)	1.49 (1.34–1.65, *P* < .001)
	Unknown	2703 (12.2%)	2663 (20.2%)		
Grade	Well	1023 (4.6%)	185 (1.4%)		
	Moderately	5138 (23.3%)	2165 (16.4%)	2.33 (1.98–2.75, *P* < .001)	2.02 (1.69–2.42, *P* < .001)
	Poorly	10,704 (48.5%)	6806 (51.5%)	3.52 (3.00–4.12, *P* < .001)	2.56 (2.14–3.05, *P* < .001)
	Undifferentiated	307 (1.4%)	137 (1%)	2.47 (1.91–3.18, *P* < .001)	2.16 (1.62–2.87, *P* < .001)
	Unknown	4911 (22.2%)	3914 (29.6%)		
GC type	Diffuse GC	16,727 (75.7%)	9675 (73.3%)		
	Intestinal GC	5356 (24.3%)	3532 (26.7%)	1.14 (1.09–1.20, *P* < .001)	0.97 (0.91–1.03, *P* = .269)
T_stage					
	Tis/T1/T2	8593 (38.9%)	2829 (21.4%)		
	T3/T4	9644 (43.7%)	4355 (33%)	1.37 (1.30–1.45, *P* < .001)	1.10 (1.03–1.17, *P* = .003)
	Unknown	3846 (17.4%)	6023 (45.6%)		
N_stage	No	10,635 (48.2%)	4514 (34.2%)		
	Yes	8664 (39.2%)	5382 (40.8%)	1.46 (1.39–1.54, *P* < .001)	1.61 (1.52–1.71, *P* < .001)
	Unknown	2784 (12.6%)	3311 (25.1%)		
Size	<3 cm	5196 (23.5%)	1286 (9.7%)		
	>3 cm	9135 (41.4%)	4157 (31.4%)	1.83 (1.71–1.97, *P* < .001)	1.65 (1.52–1.78, *P* < .001)
	Unknown	7734 (35.1%)	7764 (58.7%)		

### 3.3. Comparison of distant metastasis site between early-onset and late-onset GC

**Figure 1.**
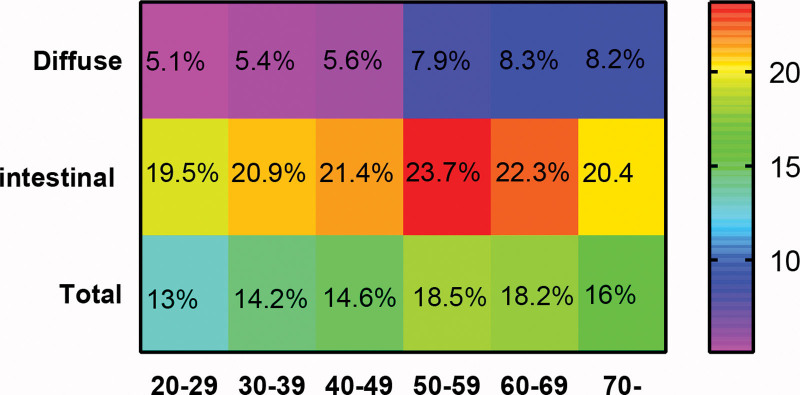
The different liver metastasis rate of different age grouping based on Lauren type.

To address the imbalanced basic information that can influence distant metastasis, we selected 3276 early-onset GC patients matched with 3276 late-onset GC patients. As displayed in Table 3, we achieved balance across the previous basic information based on the *P* value and standardized mean difference (SMD) values. Subsequently, through univariate logistic regression analysis, we discovered that early-onset GC patients exhibited a higher rate of distant metastasis compared to late-onset GC patients (53.3% vs 39.9%, *P* < .001). Further exploration focusing on various metastatic sites revealed that early-onset GC was a risk factor for bone, brain, lung, and other site metastases. Interestingly, early-onset GC patients were found to be a protective factor against liver metastasis when compared to the late-onset GC group (*P* = .0037). To delve deeper into this intriguing outcome, we conducted a multivariate logistic regression analysis. In Table 4, contrasting the results of risk factors for distant metastasis, we observed some inverse findings. Notably, late-onset GC emerged as an independent risk factor for liver metastasis (OR = 1.16, *P* = .027). Additionally, Black patients appeared more prone to liver metastasis (OR = 1.28, *P* < .001), while female patients stood out as independent protective factors against liver metastasis (OR = 0.78, *P* < .001). Factors such as poorer cell differentiation, positive LNM, and larger tumor size continued to be associated with increased risks for liver metastasis. Interestingly, we discovered that the diffuse type of GC acted as a protective factor for liver metastasis when compared to the intestinal-type of GC. Surprisingly, advanced T stage did not show an association with liver metastasis, indicating that liver metastasis may have unique characteristics distinct from metastases in other sites. Moreover, we segmented age into 6 groups (20–29, 30–39, 40–49, 50–59, 60–69, and over 70 years) and analyzed the rates of liver metastasis across these age groups. As illustrated in Figure 1, the rate of liver metastasis in diffuse-type GC increased with age, whereas in the intestinal type, it only increased up to 49 years old. Overall, liver metastasis rates rose with age in the 20 to 59 age bracket but decreased in the 60 to 69 and over 70 groups.

**Table 3:** The association of age with distant metastasis after adjusting other basic information.

	Overall	Younger GC	Older GC	*P* value	SMD value
n	6552	3276	3276		
Race (%)				.073	0.019
White	4245 (64.8)	2171 (66.3)	2074 (63.3)		
Black	847 (12.9)	399 (12.2)	448 (13.7)		
Other	1311 (21.4)	681 (20.8)	723 (22.1)		
Unknown	56 (0.8)	25 (0.7)	31 (0.9)		
Sex (%)				.319	0.065
Male	3764 (57.4%)	1862 (56.8)	1902 (58.1)		
Female	2788 (42.6)	1414 (43.2)	1374 (41.9)		
Site (%)				.096	0.031
Cardia	1312 (20.0)	664 (20.3)	648 (19.8)		
Fundus	345 (5.3)	151 (4.6)	194 (5.9)		
Body	822 (12.5)	375 (11.4)	447 (13.6)		
Antrum	1128 (17.2)	579 (17.7)	549 (16.8)		
Pylorus	294 (1.5)	165 (5.0)	129 (3.9)		
Lesser curvature	573 (8.7)	238 (7.3)	335 (10.2)		
Greater curvature	286 (4.4)	138 (4.2)	148 (4.5)		
Overlapping	742 (11.3)	362 (9.2)	380 (11.6)		
Unknown	1150 (17.6)	604 (18.4)	546 (16.7)		
Grade (%)				.305	0.067
Well-differentiated	130 (2.0)	58 (1.8)	72 (2.2)		
Moderately differentiated	753 (11.5)	367 (11.2)	386 (11.8)		
Poorly differentiated	4038 (61.6)	2051 (62.6)	1987 (60.7)		
Undifferentiated	108 (1.6)	47 (1.4)	61 (1.9)		
Unknown	1513 (23.1)	753 (23.0)	760 (23.2)		
GC type	2995 (45.7)	1497 (45.7)	1498 (45.7)	1	0.001
Intestinal type	3557 (54.3)	1779 (54.3)	1778 (54.3)		
Diffuse type	2995 (45.7)	1497 (45.7)	1498 (45.7)		
T stage (%)				.058	0.016
Tis	12 (0.2)	6 (0.2)	6 (0.2)		
T1	1122 (17.1)	547 (16.7)	575 (17.6)		
T2	696 (10.6)	329 (10.0)	367 (11.2)		
T3	1504 (23.0)	771 (23.5)	733 (22.4)		
T4	1480 (22.6)	783 (23.9)	697 (21.3)		
Unknown	1738 (26.5)	840 (25.6)	898 (27.4)		
N stage (%)				.161	0.087
No	2423 (37.0)	1176 (35.9)	1247 (39.0)		
N1	1552 (23.7)	784 (27.0)	768 (20.4)		
N2	618 (9.4)	318 (9.7)	300 (9.2)		
N3	682 (10.4)	366 (11.2)	316 (9.6)		
Unknown	1277 (19.5)	632 (19.3)	645 (19.8)		
M stage (%)				<.001	
No	3497 (53.4)	1529 (46.7)	1968 (60.1)		
Yes	3055 (46.6)	1747 (53.3)	1308 (39.9)		
Size				.642	0.089
<3 cm	1255 (19.2)	633 (19.3)	622 (19.0)		
≥3 cm	2493 (38.0)	1228 (37.5)	1265 (38.6)		
Unknown	2804 (42.8)	1415 (43.2)	1389 (42.4)		
Bone_M (%)				<.001	0.171
No	5690 (86.8)	2797 (85.4)	2893 (88.3)		
Yes	442 (6.7)	289 (8.8)	153 (4.7)		
Unknown	420 (6.4)	190 (5.8)	230 (7.0)		
Brain_M (%)				.011	
No	6063 (92.5)	3042 (92.9)	3021 (92.2)		
Yes	60 (0.9)	39 (1.2)	21 (0.6)		
Unknown	429 (6.5)	195 (6.0)	234 (7.1)		
Liver_M (%)				.0037	
No	5221 (79.7)	2630 (80.3)	2591 (79.1)		
Yes	1026 (15.7)	474 (14.5)	552 (16.8)		
Unknown	305 (4.6)	172 (5.3)	133 (4.1)		
Lung_M (%)				.001	
No	5719 (87.3)	2866 (87.5)	2853 (87.1)		
Yes	392 (6.0)	219 (6.7)	173 (5.3)		
Unknown	441 (6.7)	191 (5.8)	250 (7.6)		
Other site_M (%)	1618 (24.7)	962 (29.4)	656 (20.0)	<.001	
No	4934 (75.3)	2314 (70.6)	2620 (80.0)		
Yes	1618 (24.7)	962 (29.4)	656 (20.0)		

**Table 4:** Cross-sectional associations between other clinical factors and liver metastasis.

Dependent: liver_M		No (N = 27,270)	Yes (N = 5734)	OR (univariable)	OR (multivariable)
Age					
	Younger	2845 (10.4%)	501 (8.7%)		
	Older	24,425 (89.6%)	5233 (91.3%)	1.22 (1.10–1.34, *P* < .001)	1.16 (1.09–1.29, *P* = .0278)
Race					
	White	19,174 (70.3%)	4172 (72.8%)		
	Black	3300 (12.1%)	791 (13.8%)	1.10 (1.01–1.20, *P* = .025)	1.28 (1.17–1.41, *P* < .001)
	Other	4640 (17%)	742 (12.9%)	0.73 (0.68–0.80, *P* < .001)	0.84 (0.77–0.93, *P* < .001)
	Unknown	156 (0.6%)	29 (0.5%)		
Sex					
	Male	17,185 (63%)	4079 (71.1%)		
	Female	10,085 (37%)	1655 (28.9%)	0.69 (0.65–0.74, *P* < .001)	0.78 (0.73–0.84, *P* < .001)
Site					
	Cardia	8965 (32.9%)	2366 (41.3%)		
	Fundus	921 (3.4%)	285 (5%)	1.17 (1.02–1.35, *P* = .026)	1.29 (1.11–1.50, *P* = .001)
	Body	2572 (9.4%)	469 (8.2%)	0.69 (0.62–0.77, *P* < .001)	0.88 (0.78–0.99, *P* = .035)
	Antrum	5292 (19.4%)	740 (12.9%)	0.53 (0.48–0.58, *P* < .001)	0.71 (0.64–0.78, *P* < .001)
	Pylorus	762 (2.8%)	100 (1.7%)	0.50 (0.40–0.62, *P* < .001)	0.65 (0.52–0.82, *P* < .001)
	Lesser curvature	2123 (7.8%)	321 (5.6%)	0.57 (0.51–0.65, *P* < .001)	0.76 (0.67–0.88, *P* < .001)
	Greater curvature	943 (3.5%)	172 (3%)	0.69 (0.58–0.82, *P* < .001)	0.94 (0.78–1.12, *P* = .479)
	Overlapping	2082 (7.6%)	384 (6.7%)	0.70 (0.62–0.79, *P* < .001)	0.94 (0.82–1.07, *P* = .324)
	Unknown	3610 (13.2%)	897 (15.6%)		
Grade					
	Well	1058 (3.9%)	103 (1.8%)		
	Moderately	5570 (20.4%)	1380 (24.1%)	2.54 (2.06–3.14, *P* < .001)	2.16 (1.74–2.69, *P* < .001)
	Poorly	13,960 (51.2%)	2613 (45.6%)	1.92 (1.56–2.36, *P* < .001)	2.02 (1.62–2.51, *P* < .001)
	Undifferentiated	371 (1.4%)	51 (0.9%)	1.41 (0.99–2.02, *P* = .057)	1.83 (1.26–2.67, *P* = .002)
	Unknown	6311 (23.1%)	1587 (27.7%)		
GC type					
	Intestinal GC	19,455 (71.3%)	5186 (90.4%)		
	Diffuse GC	7815 (28.7%)	548 (9.6%)	0.26 (0.24–0.29, *P* < .001)	0.27 (0.24–0.30, *P* < .001)
T stage					
	Tis/T1/T2	9930 (36.4%)	1261 (22%)		
	T3/T4	12,196 (44.7%)	1551 (27%)	1.00 (0.93–1.08, *P* = .971)	
	Unknown	5144 (18.9%)	2922 (51%)		
N stage					
	No	13,119 (48.1%)	1831 (31.9%)		
	Yes	11,452 (42%)	2352 (41%)	1.47 (1.38–1.57, *P* < .001)	1.69 (1.57–1.83, *P* < .001)
	Unknown	2699 (9.9%)	1551 (27%)		
Size					
	<3 cm	26,048 (95.5%)	4927 (85.9%)		
	≥3 cm	1127 (4.1%)	590 (10.3%)	2.77 (2.49–3.07, *P* < .001)	1.83 (1.63–2.06, *P* < .001)
	Unknown	95 (0.3%)	217 (3.8%)		

## 4. Discussion

In clinical practice, the Lauren classification is widely used. According to a previous study, there has been an observed increase in the incidence of diffuse-type GC over the past decades, while the incidence of intestinal-type GC has seen a decrease.^[10]^ A similar trend of increased incidence has been noted in early-onset GC patients (under 50 years old). This indicates that both early-onset GC patients and those with diffuse-type GC should receive heightened attention and focus in clinical settings.^[10]^ In our study leveraging a large population database, we conducted an analysis of the clinical characteristics between early-onset GC and late-onset GC. Our findings suggested that early-onset GC demonstrated a higher degree of malignantly invasive ability overall. Interestingly, when focusing specifically on liver metastasis, we observed that early-onset GC exhibited a weaker invasive capacity compared to late-onset GC, where the latter displayed greater invasive potential in this context.

In our study, we established the age cutoff at 50 years, categorizing patients under this threshold as early-onset GC or early-onset GC. This approach aligns with the methodology employed in other studies in the field.^[8,11]^ Of course, some studies considered GC patients under 45 years old at diagnosis as early-onset GC.^[12,13]^ The onset of GC is primarily concentrated between the ages of 50 and 70, with the incidence rate showing an upward trend as age increases. Given the higher risk of long-term health issues and the rising burden of cancer among young adults, the US National Cancer Institute has labeled this phenomenon an early-onset cancer epidemic.^[14]^ Actually, the differences in epidemiology and clinical, pathological, and molecular characteristics clearly exist between early-onset and late-onset cancers, however, these differences do not change dramatically at exactly 50 years older.^[15]^ It is a limitation of applying the cutoff point of 50 years older. In a recent review, the definition of early-onset GC was defined as patients under 50.^[14]^ Different studies have different definitions of early-onset GC, including considering patients who develop the disease before the age of 40 or 45 as having early-onset GC.^[16–18]^ The majority of studies still consider the age of 50 as the cutoff point for early-onset GC.^[8,19]^

Despite variations in age cutoff values, our study outcomes are consistent with prior research findings. Notably, we believe our study is the first to comprehensively delineate the characteristics of distant metastasis. While limited studies have delved into the realm of early-onset GC and Lauren type, congruent with our results, some existing studies have highlighted that early-onset GC patients exhibit more aggressive clinicopathological features. These may include a higher prevalence of tumors with positive LNM, advanced T stages, and poorer cell differentiation.^[8,13,20]^ The increasing incidence of early-onset GC shows to be a worrisome trend, which is correlated with many etiologies such as genomic alteration, lifestyle, and environmental factors.^[21]^ Managing early-onset GC patients poses a distinctive challenge due to several unknown traits and pathogenetic factors. Our study encompassed a total of 36,973 GC patients, with 3684 categorized as early-onset patients and 33,289 as late-onset patients. Analysis of basic information unveiled that the proportion of female patients, tumors located in the gastric body, and the prevalence of diffuse-type GC were higher among early-onset GC patients compared to late-onset GC patients. The observed increase in the incidence of female patients among early-onset GC individuals aligns with findings reported in many other studies as well.^[13,18]^ The larger rate of diffuse-type GC and poorer differentiated GC was in younger GC compared to older GC, which was referred to in some studies.^[8,22]^

In our investigation into distant metastasis, we observed that the overall positive metastasis rate for GC was 35.7%. Among these cases, the rates of metastasis to bone, brain, lung, liver, and other sites were found to be 4.8%, 0.7%, 5.3%, 15.5%, and 14.7%, respectively. Corresponding with our findings, other studies have similarly highlighted that liver and abdominal cavity involvement are common routes of distant metastasis in GC.^[23,24]^ Tumors located in different sites within the stomach exhibited varying rates of distant metastasis. In comparison to tumors situated in the cardia, those found in the fundus and body regions of the stomach were identified as risk factors for distant metastasis, while tumors in the antrum and pylorus regions were protective factors against distant metastasis. These findings are likely associated with the distribution patterns of lymph nodes and vessels in these particular regions.^[25]^ Moreover, genomic alterations vary across different regions of the stomach, leading to distinct patterns of metastasis. For example, the CIN subtype predominantly manifests in GCs situated in the cardia, whereas MSI tumors are most commonly found in the noncardia region of the stomach. EBV tumors, on the other hand, tend to occur most frequently in the fundus or body of the stomach. Liver metastasis in GC involves a multifaceted process that includes direct invasion, lymphatic vessel invasion, and hematogenous spread. A retrospective study has demonstrated that liver metastasis significantly impacts the 5-year survival rate and serves as an independent prognostic factor for GC patients with adjacent organ invasion. This highlights the potential benefit of combined organ resection in such cases.^[26]^ Additionally, curability resection for GC with liver metastasis has an overall 5-year survival rate of more than 30%.^[25]^ Identifying and assessing liver metastasis in GC early on is crucial to mitigate bodily harm and enhance patient prognosis. In our study, we observed that early-onset age based on Lauren type served as a protective factor against liver metastasis. Through multivariate logistic analysis, we determined that Lauren type did not exhibit a significant association with overall distant metastasis but emerged as an independent risk factor specifically for liver metastasis. Notably, diffuse-type GC carried a lower risk of liver metastasis. Therefore, we propose that the increased proportion of diffuse-type cases among early-onset GC patients may explain why early-onset individuals demonstrate reduced instances of liver metastasis. Several studies have explored the link between age and liver metastasis. For instance, An et al discovered no discernible relationship between age and liver metastasis after categorizing age groups into < 60 and ≥ 60 years old.^[27]^ Lin et al segmented age into multiple groups, which aligns with our approach. They similarly concluded that age did not exhibit a correlation with liver metastasis. This lack of association was attributed to the fact that Lin et al study focused on all types of GC rather than specifically examining the Lauren classification like in our research.^[24]^ The lower occurrence of liver metastasis in diffuse GC can be attributed to genomic variances between diffuse and intestinal types. Factors such as CDH1 expression, immunoproteasome subunits, and microsatellite instability differ between these types, influencing their respective invasive capabilities. These genomic distinctions play a crucial role in shaping the propensity for metastasis in each GC subtype.^[28–30]^ CDH1 mutation is the most frequent genetic alteration which is highly associated with early-onset diffuse GC.^[31]^ A previous study unveiled that intestinal-type GC and late-onset patients were often linked with a high frequency of TP53 mutations, predisposing them to liver metastasis. Conversely, diffuse-type GC exhibited a higher incidence of chromosomal stability, which was correlated with a tendency toward peritoneal metastasis. These distinct genetic characteristics play a pivotal role in determining the metastatic patterns observed in different types of GC.^[32]^

It is important to note that our study has several limitations inherent to the use of SEER data. These include potential biases related to data completeness, accuracy of coding, and missing information on certain clinical variables. Additionally, the retrospective nature of the study limits our ability to establish causal relationships or account for confounding factors that could influence the observed associations between age at diagnosis and GC distant metastasis. In our study, we did not incorporate additional clinical characteristics like lymphatic vessels and treatments, which could potentially influence distant metastasis. Moreover, our examination of distant metastasis patterns was restricted to liver, bone, brain, and lung metastases, thus presenting a limitation. Peritoneal and ovarian metastases were not explored in our study, further highlighting a constraint in our findings.

## 5. Conclusions

In conclusion, our study provided a comprehensive examination of factors associated with distant metastasis in GC, encompassing age, tumor size, chemotherapy, T stage, and N stage. We identified early-onset age as a protective factor against liver metastasis in GC, potentially linked to the higher prevalence of diffuse-type GC among early-onset patients. The specific mechanisms underlying the lower occurrence of liver metastasis in early-onset GC individuals warrant further exploration through future clinical and basic research endeavors.

## Author contributions

**Conceptualization:** Feng Wu.

**Data curation:** Shujie Shuai.

**Formal analysis:** Shuang Wu, Shujie Shuai.

**Methodology:** Shuang Wu.

**Project administration:** Feng Wu.

**Software:** Feng Wu, Shuang Wu.

**Supervision:** Feng Wu, Shujie Shuai.

**Validation:** Shujie Shuai.

**Writing – original draft:** Feng Wu.
